# Self‐Microemulsifying Drug Delivery System: Liquid‐Filled Gelatin Capsules of Atorvastatin and Ezetimibe

**DOI:** 10.1155/adpp/8642343

**Published:** 2026-11-04

**Authors:** Yara Nabeel Al-Salhi, Hani Naseef, Moammal Qurt, Abdallah Damin Abukhalil, Abdullah K. Rabba, Israr Sabri, Fuad Al-Rimawi

**Affiliations:** ^1^ Pharmacy Department Faculty of Pharmacy, Nursing and Health Professions Birzeit University P.O. Box 14 Birzeit West Bank State of Palestine birzeit.edu; ^2^ Department of Chemistry at Al-Quds University Abu Dis State of Palestine

**Keywords:** atorvastatin, ezetimibe, microemulsion, self-microemulsifying

## Abstract

Atorvastatin (ATV) and ezetimibe (EZE) are BCS Class II drugs with low oral bioavailability (14% and 35%, respectively) attributed to poor aqueous solubility and extensive first‐pass metabolism. Although the two drugs act synergistically to lower cholesterol, their combination into a single delivery system is complicated by differing physicochemical properties. This study aimed to develop and characterize a self‐microemulsifying drug delivery system (SMEDDS) coloaded with ATV and EZE, encapsulated in an Empo Caps LP + size 00 capsule, to improve their dissolution and permeability. Pseudoternary phase diagrams were constructed to identify microemulsion regions, and candidate formulations were screened by droplet size, polydispersity index, refractive index, and viscosity. Four formulations (ME#22, ME#26, ME#31, and ME#35) met the physical acceptance criteria, including a polydispersity index (PDI) within its theoretically valid 0–1 range; a fifth trial (ME#12) was excluded on this basis. Formula 31—containing 14.96% triacetin, 39.89% Tween 80%, and 19.94% PEG 400 (oil:surfactant/cosurfactant ratio 2:8; surfactant:cosurfactant ratio 2:1)—showed the highest drug loading (ATV 3.95 ± 0.35 mg/mL; EZE 3.04 ± 0.06 mg/mL), a droplet size of 74.15 ± 1.68 nm, PDI of 0.34 ± 0.02, refractive index of 1.372, and viscosity of 310 ± 9.58 cP, with assay values of 104.6% (ATV) and 104.9% (EZE). This formulation demonstrated faster in vitro dissolution than the commercial reference product (Atozet) across all tested media and significantly greater ex vivo intestinal permeability than plain drug suspension (*p* < 0.05 from 90 min onward). In conclusion, an ATV‐/EZE‐loaded SMEDDS encapsulated in an Empo Caps LP + size 00 capsule was successfully developed and showed improved in vitro dissolution and ex vivo permeability relative to the marketed product, supporting its potential as an alternative oral delivery strategy for this drug combination.

## 1. Introduction

Dyslipidemia, characterized by elevated low‐density lipoprotein cholesterol (LDL‐C), is a major modifiable risk factor for atherosclerotic cardiovascular disease (ASCVD), the leading cause of morbidity and mortality worldwide [1]. Current management combines lifestyle modification—increased physical activity and a diet low in saturated fat—with pharmacotherapy in patients at elevated risk [1–3]. Statins such as atorvastatin (ATV) remain first‐line therapy, and combination with a cholesterol‐absorption inhibitor such as ezetimibe (EZE) provides a complementary, synergistic reduction in LDL‐C beyond what either agent achieves alone.

Oral administration remains the preferred route for chronic cardiovascular therapy because of its convenience and ease of use; however, its effectiveness depends on adequate aqueous solubility and intestinal permeability of the active drug [2]. ATV and EZE are both Biopharmaceutical Classification System (BCS) Class II compounds, characterized by poor aqueous solubility and, consequently, low and variable oral bioavailability (14% and 35%, respectively) [4, 5]. Improving the dissolution behavior of these drugs is therefore a key pharmaceutical challenge in developing an effective combined oral therapy.

Lipid‐based delivery systems, particularly microemulsions, are a well‐established strategy for improving the oral delivery of poorly water‐soluble, lipophilic drugs [6]. A microemulsion is a thermodynamically stable, isotropic mixture of oil, water, surfactant, and cosurfactant, with droplet sizes typically ranging from 10 to 200 nm; dissolving a lipophilic drug within the oil phase increases its effective solubility and improves dissolution in gastrointestinal fluids [6, 7]. A self‐microemulsifying drug delivery system (SMEDDS) is a related, anhydrous formulation—an isotropic mixture of oil, surfactant, and cosurfactant—that spontaneously forms a fine oil‐in‐water microemulsion upon mild agitation in aqueous or gastrointestinal fluids, a process termed in situ self‐emulsification [8, 9]. Because SMEDDS presents the drug already dissolved and finely dispersed prior to dilution, they can enhance solubilization and dissolution rate, which may in turn support improved oral bioavailability [8].

ATV is a weak acid (pKa 4.6) with pH‐dependent solubility that is markedly reduced under acidic gastric conditions, while EZE is a weakly acidic (phenolic hydroxyl, pKa 9.75), practically water‐insoluble compound that remains predominantly unionized across the physiological gastrointestinal pH range [10, 11]. The two drugs also differ in their preferred formulation environment: ATV calcium is more chemically stable in basic media, which suppresses lactonization, whereas basic conditions promote the degradation of EZE [10]. These divergent physicochemical and stability requirements make coformulating ATV and EZE in a single delivery system technically challenging, and existing combination products—such as bilayer tablets separating ATV as an immediate‐release layer from EZE as a sustained‐release layer—address this primarily through physical rather than solubility‐based separation [12].

Liquid‐filled hard gelatin capsules, such as Empo Caps LP+, are a liquid‐ or semisolid‐fill dosage form that can encapsulate a preformed SMEDDS directly, avoiding the additional processing steps (e.g., granulation, drying) required to convert a liquid SMEDDS into a solid oral dosage form. Because the drug is already solubilized within the fill matrix at the time of manufacture, the dissolution of the capsule shell can expose the formulation to gastrointestinal fluid rapidly; however, the extent to which this translates into improved bioavailability depends on both the fill formulation and the properties of the incorporated drug, rather than being an inherent property of the capsule shell itself [13, 14].

Although SMEDDS formulations of ATV and EZE have been individually reported, and a solid self‐nanoemulsifying system combining the two drugs has been described [15], to our knowledge no prior study has coformulated ATV and EZE within a single liquid SMEDDS encapsulated in an Empo Caps LP + size 00 capsule using this excipient combination (triacetin, Tween 80, and PEG 400). This work therefore aimed to develop and characterize an ATV/EZE‐loaded SMEDDS‐filled Empo Caps LP + size 00 capsule, using pseudoternary phase diagrams to identify suitable oil–surfactant–cosurfactant combinations and to evaluate the optimized formulation’s physicochemical properties, in vitro dissolution, and ex vivo intestinal permeability relative to a marketed combination tablet.

## 2. Materials and Methods

### 2.1. Chemicals

ATV calcium and EZE were kindly donated by Birzeit University and Birzeit Pharmaceutical Company (Birzeit, State of Palestine). Kolliphor RH 40 was obtained from BASF SE (Ludwigshafen, Germany); Tween 80 and ethyl oleate from Croda International Plc (Snaith, UK); Tween 20 from Merck KGaA (Darmstadt, Germany); propylene glycol (PG) from Dow Europe GmbH (Horgen, Switzerland); polyethylene glycol 400 from The Dow Chemical Company (Midland, MI, USA); oleic acid from Fisher Chemical (Fisher Scientific, Loughborough, UK); castor oil, olive oil, and soybean oil from Gustavees (full company name/location to be confirmed); and triacetin (99%) from Thermo Fisher Scientific (Waltham, MA, USA). Acetonitrile, methanol, and ammonium acetate buffer were purchased from Sigma‐Aldrich (St. Louis, MO, USA).

### 2.2. Development of Analytical Method

The ATV/EZE combination was assayed using an in‐house developed and validated high‐performance liquid chromatography (HPLC) system (Dionex, 12000 series; Thermo Fisher Scientific) equipped with a UV detector. Method validation (Section 3.1) was performed using a Hypersil ODS C18 column (4.6 × 250 mm, 5‐μm particle size; Illinois, USA); routine sample analysis throughout this study was subsequently performed using a YMC C18 column of equivalent dimensions and particle size (4.6 × 250 mm, 5 μm). The mobile phase consisted of 0.1 M ammonium acetate buffer and acetonitrile (2:3, v/v), adjusted to pH 6.0 with phosphoric acid and filtered under vacuum prior to use. Both drugs were detected at a single shared wavelength of 250 nm, rather than at each drug’s individually optimal wavelength (commonly reported in the literature as approximately 246 nm for ATV and 232 nm for EZE when assayed separately) [16, 17]. A single compromise wavelength is standard, well‐precedented practice specifically for simultaneous combination assays of this drug pair using a single‐wavelength UV detector (as used in this study, rather than a multiwavelength PDA detector): published, validated simultaneous ATV/EZE HPLC methods have reported single detection wavelengths of 240 nm (as also cited in Refs. [17–19]), 251, 228, and 254 nm, illustrating that a shared, near‐isosbestic compromise wavelength—rather than each drug’s individual absorption maximum—is the conventional approach for this class of method, since it avoids the need for wavelength switching or a second detector channel during a single chromatographic run. Our selected wavelength (250 nm) falls within this same established range, close to the 251 nm reported by at least one prior validated method for this exact drug combination [18]. The flow rate was 1.0 mL/min, column temperature was maintained at 25°C, and the injection volume was 20 μL. Samples were filtered through a 0.45‐μm‐membrane filter, diluted first in methanol and then in mobile phase, and analyzed by HPLC [17–19].

Method validation was conducted according to ICH guideline Q2 (R2) [19], assessing linearity and range, system precision, method precision, accuracy, specificity (forced degradation and placebo interference), robustness, ruggedness, and the limit of detection (LOD) and limit of quantification (LOQ), the latter two determined from a dedicated calibration curve spanning 50–200 ppm (Section 3.1). System suitability acceptance criteria were as follows: relative standard deviation (RSD) not more than 2.0%, column efficiency not less than 2000 theoretical plates, resolution between ATV and EZE peaks not less than 3.0, and a tailing factor between 0.8 and 2.0. Full validation results are reported in Section 3.1.

### 2.3. Evaluation of ATV and EZE Solubility

An excess quantity (200 mg) of ATV and EZE, respectively, was added to 2 mL of each selected excipient in a screw‐cap tube. The mixture was capped and vortex‐mixed for 5 min, and then agitated in a shaking water bath at 150 rpm and 40°C for 72 h to reach saturation equilibrium. A temperature of 40°C was selected to moderately accelerate the attainment of equilibrium solubility relative to room temperature, while remaining well below temperatures that could risk thermal degradation of the incorporated actives, consistent with the range of temperatures (25°C–55°C) reported for similar saturation solubility studies in the SMEDDS/SEDDS literature. Equilibrium was confirmed by the absence of further visible precipitate change and consistent drug readings at the final sampling timepoints. Each tube was then centrifuged at 5000 rpm for 10 min, and the supernatant was filtered through a 0.45‐μm membrane filter, diluted appropriately, and analyzed by the validated HPLC method (Section 2.2) to determine drug solubility (mg/mL). All experiments were performed in triplicate (*n* = 3), and the results are reported as mean ± SD.

### 2.4. Construction of Pseudo Ternary Phase Diagram

Pseudoternary phase diagrams were constructed separately for each oil phase (triacetin, oleic acid, sesame oil, castor oil, olive oil, and soybean oil) to identify the microemulsion region for each oil–surfactant–cosurfactant system. For each diagram, the surfactant and cosurfactant were combined at fixed weight ratios (Smix) of 1:1, 1:2, 2:1, 1:3, and 3:1. Each Smix was then blended with the corresponding oil at nine oil:Smix weight ratios ranging from 1:9 to 9:1, and the mixtures were vortexed to homogeneity. Distilled water was used for the titration (rather than tap water) to avoid dissolved ions and mineral content that could interfere with surfactant self‐assembly and phase behavior. Rather than a continuous drop‐by‐drop titration, water was added in discrete, stepwise increments calculated to raise the cumulative water content of the total system by 5% (w/w) at each step (i.e., 5%, 10%, 15%, 20%, and so on). After each addition, the mixture was vortexed to homogeneity and visually assessed against a light source for clarity, and classified as clear (monophasic, within the microemulsion‐forming region) or turbid/phase‐separated. This stepwise process was repeated, increasing the cumulative water content in successive 5% (w/w) increments, until the mixture was no longer visibly clear or until a defined upper bound of water content (up to 70% w/w) was reached without loss of clarity, whichever occurred first. The maximum cumulative water content at which the system remained clear was recorded as that trial’s endpoint, and the corresponding phase boundaries were plotted as pseudoternary phase diagrams using SigmaPlot [20].

### 2.5. Preparation of ATV‐ and EZE‐Loaded SMEDDS

**TABLE 1:** Water tolerance (% w/w) at the monophasic/clear transition point, by oil (mean ± SD).

Oil	*n* (trials)	Mean water tolerance (%)	SD
Triacetin	26	43.1	21.4
Oleic acid	17	17.4	4.7

These blank (drug‐free) trial formulations—oil, surfactant, cosurfactant, and water combined directly according to each selected ternary diagram point—were therefore genuine, water‐containing microemulsions, not an anhydrous concentrate, and were characterized directly in this form for viscosity, refractive index, droplet size, and dye miscibility testing (Section 2.6).

Separately, for drug loading and subsequent capsule filling (Section 2.7), an anhydrous SMEDDS concentrate was prepared for the selected formulations. The surfactant and cosurfactant were combined and mixed using a magnetic stirrer at 300–600 rpm at room temperature, consistent with mixing speeds reported for SMEDDS/microemulsion preparation elsewhere in the literature [21], followed by the addition of the oil phase, with continuous mixing until a clear, homogeneous anhydrous mixture was obtained.

ATV and EZE were then added to this anhydrous SMEDDS base, in amounts equivalent to 40 mg ATV and 10 mg EZE per unit dose, and mixed at room temperature until a visually clear, drug‐loaded SMEDDS was obtained, with no undissolved drug particles observed.

This anhydrous, drug‐loaded SMEDDS is the material used for capsule filling (Section 2.7) and is designed to self‐emulsify upon contact with gastrointestinal fluid. The water‐containing blank trial formulations (Table 1) served specifically to characterize and screen the underlying oil–surfactant–cosurfactant–water phase behavior prior to this stage [20, 22, 23].

### 2.6. Characterization and Evaluation Tests

After the preparation of the microemulsions, they were subjected to the following characterization and evaluation tests.

#### 2.6.1. Viscosity Measurement

Viscosity was measured using a Brookfield DV2T viscometer fitted with a B‐LV‐1 (spindle 61) spindle, selected on the basis of the expected low‐to‐moderate viscosity range of the formulations (12–310 cP), consistent with Brookfield’s standard guidance that spindle size be matched to sample viscosity to obtain readings within the reliable torque range of the instrument, and consistent with spindle selection reported for comparable low‐viscosity pharmaceutical formulations [21]. Measurements were taken at 25°C (standard ambient laboratory temperature, consistent with conditions reported for comparable microemulsion viscosity studies). Viscosity was assessed at four rotation speeds (10, 20, 50, and 100 rpm); readings were successfully recorded at 50 and 100 rpm for all formulations, and these two speed‐based readings are reported here as mean ± SD (Table 1), while the viscometer could not read the readings at 10 and 20 rpm. Shear rate at each recorded rotation speed was calculated from the standard Brookfield spindle‐specific shear rate constant for spindle 61. Viscosity was measured because it is a required input parameter for calculating droplet size by dynamic light scattering (DLS) (via the Stokes–Einstein relationship; Section 2.6.3).

#### 2.6.2. Refractive Index Measurement

The refractive index of each formulation was measured using a refractometer (KRUSS Optronic GmbH, model DR6000‐T) at 25°C, with each measurement performed in triplicate and reported as mean ± SD, consistent with the temperature and replicate conditions reported for the refractive index testing of comparable microemulsion systems [24]. The refractive index was measured for two reasons: First, because microemulsions are optically isotropic and transparent, refractive index values close to that of water (1.3316) confirm the transparency and homogeneity of each formulation trial; and second, the refractive index is a required input parameter for the droplet size and polydispersity index calculation by DLS (Section 2.6.3).

#### 2.6.3. Droplet Size and Size Distribution

Mean droplet size and polydispersity index were determined by DLS using a Particle Sizer and Zeta Potential Analyzer (NanoBrook Omni, Brookhaven Instruments), measured directly on the water‐containing blank trial formulations described in Section 2.5 (Table 1), which already incorporate water as a formulated component per the ternary diagram composition for each trial. Polydispersity index (PDI) was calculated automatically by the instrument software via cumulants analysis of the measured intensity autocorrelation function (per ISO 13321/22412), reported as the normalized second cumulant, which describes the width of the fitted particle size distribution relative to the mean size. This method assumes an approximately Gaussian, unimodal size distribution and becomes progressively less reliable for PDI values above approximately 0.3 (see Section 4.3 for the implications of this for formulation ME#12, which had a PDI of 1.76). Measurements were performed in triplicate and reported as mean ± SD.

#### 2.6.4. Thermodynamic Stability

Formulations were evaluated using a three‐stage thermodynamic stability protocol, following the general stress‐testing approach reported for microemulsion systems in the literature [25, 26]: Monophasic behavior (i.e., a single, clear, homogeneous phase with no visible turbidity, creaming, or layering) was assessed by visual inspection against both light and dark backgrounds, comparing each formulation before and after each stress test; formulations were classified as monophasic if no phase separation, turbidity, or precipitation was observed, and biphasic/failed if any such change occurred, consistent with the visual assessment approach used for the water titration/phase diagram endpoint (Section 2.4). (i) Heating–cooling cycling: six cycles of not less than 48 h each between 4°C (refrigerated) and 45°C; formulations remaining physically stable (no phase separation, creaming, or cracking) proceeded to (ii) centrifugation testing: Formulations were diluted with water and centrifuged at 4000 rpm for 45 min, within the range of centrifugation speeds and durations reported for microemulsion stress testing elsewhere [25, 26]; formulations passing this test proceeded to (iii) freeze–thaw cycling: three cycles between −4°C and +40°C, with storage for not less than 24 h at each temperature, consistent with the three‐cycle structure commonly reported for this stress test [25, 27]. Formulations passing all three thermodynamic stress tests underwent an additional short‐term checkpoint, consisting of droplet size measurement after 1 week of storage at room temperature, prior to being carried forward to drug loading (Section 2.5) and further stability/dissolution testing. Long‐term storage stability was formally assessed via the 30‐day capsule stability study described in Section 2.8; this duration is shorter than the 12‐month long‐term storage period typically recommended by ICH stability guidelines, and should be regarded as a preliminary rather than definitive long‐term stability assessment. Beyond this formal 30‐day study, the capsules were retained under the same storage conditions (refrigerated, 30°C, and 40°C) for approximately 12 months during the completion of this work; however, these capsules were not re‐assayed or re‐examined at any later timepoint, and no further stability data are therefore available or claimed for this extended period [22].

#### 2.6.5. Dye Miscibility Test

This test was used to determine whether the resulting emulsion was oil‐in‐water (o/w) or water‐in‐oil (w/o), using the following predefined, objective evaluation criteria: A water‐soluble dye (methyl orange) was added to each formulation, and the formulation was scored as o/w if the dye dispersed uniformly throughout the continuous phase within visual assessment (no localized color concentration, no precipitation) or scored as w/o if dye uptake was confined to discrete dispersed‐phase droplets, producing a visually nonuniform, speckled color distribution [15, 28]. We note that dye miscibility alone is not considered a definitive confirmatory test for continuous phase identity and is more conventionally used alongside a second, independent method such as electrical conductivity measurement (o/w systems characteristically show substantially higher conductivity than w/o systems, owing to the continuous aqueous phase); conductivity measurement was not performed in this study, and this is noted as a limitation (Section 4.3). However, the formulation strategy itself was designed specifically to favor o/w microemulsion formation: hydrophilic, high‐HLB surfactants (Tween 80, Tween 20, Kolliphor RH40) were selected throughout, and only oil:surfactant/cosurfactant ratios producing a monophasic, clear, low‐viscosity dispersion upon water titration (Section 2.4) were carried forward, consistent with standard practice for identifying the o/w‐forming region of a pseudoternary phase diagram intended for oral SEDDS/SMEDDS use; w/o‐forming regions, which are unsuitable for this application, were excluded at the phase‐diagram stage rather than encountered downstream [8, 23].

#### 2.6.6. Cloud Point and Formulation pH

Cloud point and formulation pH were not determined in this study. Cloud point testing (heating the diluted microemulsion until it turns turbid, indicating the dehydration of the surfactant’s hydrophilic head groups) is commonly used to confirm that a formulation remains thermodynamically stable and nonturbid at physiological/storage temperature, and formulation pH can influence both drug ionization/solubility and capsule shell compatibility; both are therefore recommended as core characterization tests in future work on this formulation, alongside conductivity measurement (Section 2.6.5).

#### 2.6.7. Liquid‐Filled Gelatin Capsule Preparation

Selected SMEDDS formulations (the anhydrous concentrate, not a water‐added microemulsion; see Section 2.5) were filled into Empo Caps LP + size 00 capsules. The density of each formulation was calculated from its excipient composition (oil, surfactant, and cosurfactant ratios; Table 1) using literature densities for each component (triacetin, 1.155 g/mL; Tween 80, 1.064 g/mL; Tween 20, 1.100 g/mL; PEG 400, 1.128 g/mL; PG, 1.036 g/mL) under the assumption of ideal, volume‐additive mixing. Using this density, the fill volume corresponding to a fixed target fill weight of 500 mg (equivalent to 40 mg ATV, 10 mg EZE, and 450 mg excipients) was calculated for each formulation (Table 2) and dispensed manually into the capsule shells using a calibrated pipette. The filled capsules were then sealed by hand and banded with a gelatin solution to prevent leakage of the liquid fill, a standard technique for liquid‐filled hard gelatin capsules. Fill weight uniformity was assessed by individually weighing multiple filled capsules per formulation and calculating the mean and RSD of fill weight, consistent with standard uniformity‐of‐fill‐weight testing for liquid‐/semisolid‐filled capsules (USP <905>).

**TABLE 2:** Forced degradation results by stress condition (Section 3.1).

Stress condition	API	Assay (%)	Degradation (%)
Peroxide (H_2_O_2_)	Atorvastatin	19.20	80.80
Peroxide (H_2_O_2_)	Ezetimibe	89.70	10.30
Base (NaOH)	Atorvastatin	90.0	10.0
Base (NaOH)	Ezetimibe	98.4	1.6
Acid (HCl)	Atorvastatin	19.30	80.70
Acid (HCl)	Ezetimibe	89.80	10.20

*Note:* Acceptance target: 5%–20% degradation. Ezetimibe met this target under peroxide and acid stress; atorvastatin exceeded it under both conditions (see Section 3.1 for discussion and planned corrective retesting). Heat and UV stress data are reported separately.

### 2.7. Stability Study

Stability of the blank microemulsion formulations was assessed after thermodynamic stability testing (Section 2.6.4). Drug‐loaded formulations were subsequently evaluated for assay and droplet size at 0, 1, and 2 weeks of storage at room temperature, in triplicate (*n* = 3) per timepoint. Filled capsules were stored at refrigerated conditions, 30°C/65% relative humidity, and 40°C/75% relative humidity for 30 days (*n* = 3 capsules per timepoint per condition) and assessed for drug content (assay) and physical appearance (visual inspection for leakage and phase separation). Relative humidity at each storage condition was controlled using a stability chamber and confirmed by laboratory records: 65% RH at 30°C and 75% RH at 40°C, corresponding to the standard ICH intermediate and accelerated stability storage conditions, respectively; refrigerated storage did not require RH control given the sealed, low‐temperature storage environment. This confirms and replaces an earlier draft of this manuscript, which had inferred these RH values as a reasonable assumption pending confirmation rather than as a directly confirmed experimental condition.

Formulations and capsules were considered acceptably stable if the assay remained within 95%–105% of the initial (time‐zero) value and no visible phase separation or leakage occurred; this acceptance range is consistent with typical potency/assay specifications used for pharmaceutical stability studies. The 30‐day duration used here is shorter than the minimum 3‐month period typically required by ICH stability guidelines for early phase or preliminary stability data, and this study should accordingly be regarded as a preliminary short‐term stability assessment rather than a definitive ICH‐compliant stability study; formal ICH‐compliant stability testing (≥ 3 months, controlled temperature and humidity, extended to 12 months for long‐term storage claims) is recommended before regulatory submission. Viscosity was not included in the stability monitoring panel for either the microemulsion or capsule stability studies; while assay, droplet size, and visual appearance are commonly used as core stability indicators for SMEDDS/microemulsion systems, viscosity monitoring over the storage period would provide additional information on physical stability and is recommended as an addition in future stability studies of this formulation.

### 2.8. In Vitro Drug Release Studies

Dissolution testing was performed using a USP Apparatus II (paddle) at a rotation speed of 75 rpm and 37 ± 0.5°C, consistent with the rotation speed and general method reported for a previously validated ATV/EZE combination dissolution method. Thirteen capsules were confirmed to remain fully submerged throughout testing, with no visible undissolved drug observed at any timepoint. A formal sink condition calculation was performed using published crystalline aqueous solubility values (ATV and EZE were not independently solubility‐tested in the dissolution buffers themselves in this study): At the full dose (40 mg ATV, 10 mg EZE) in 900‐mL medium, the theoretical maximum concentration (*C*_max_) was 0.044 mg/mL for ATV and 0.011 mg/mL for EZE. Using the standard sink‐condition criterion (*C*_max_ ≤ 1/3 of saturation solubility), this concentration is within a defensible margin of ATV’s reported aqueous solubility, but exceeds the corresponding threshold for EZE’s crystalline aqueous solubility (≈ 0.008 mg/mL), by approximately fourfold. However, this calculation uses the solubility of crystalline EZE in simple aqueous buffer, not the apparent solubility of EZE when solubilized within the SMEDDS/microemulsion droplets used in this formulation, which our own data (Section 3.2) show can reach several mg/mL in the formulation’s oil phase—substantially higher than the crystalline value—and is the more relevant apparent solubility during the dissolution testing of the SMEDDS‐filled capsule. No visible undissolved drug or turbidity consistent with saturation was observed during testing, consistent with sink conditions being maintained in practice; however, we did not independently confirm the apparent solubility of EZE in the dissolution buffer specifically in the presence of the solubilized formulation, and this is recommended as a specific confirmatory measurement in future work, given the crystalline‐solubility‐based calculation above technically falls outside the standard sink‐condition margin for EZE.

Dissolution studies were performed using 900 mL of dissolution medium containing 0.2% (w/v) Tween 20 (equivalent to 12 g Tween 20 dissolved in 6 L of medium) maintained at 37 ± 0.5°C. The addition of Tween 20 provided sufficient solubilizing capacity for ATV and EZE to maintain sink conditions throughout the study and minimize precipitation of the released drugs. At each sampling interval, 5‐mL aliquots were withdrawn, filtered, and analyzed by HPLC, and an equal volume (5 mL) of fresh dissolution medium preequilibrated to 37 ± 0.5°C was immediately added to maintain a constant dissolution volume and preserve sink conditions throughout the experiment [12].

Selected formulations (ME#22, ME#26, ME#31) and the Atozet brand tablet, each containing 40 mg ATV and 10 mg EZE, were tested in 900 mL of pH 6.8 phosphate buffer, the medium used for the primary formulation comparison. Formula 31 was additionally tested across the physiologically relevant gastric‐to‐intestinal pH range using three media, each 900 mL: 0.1 N HCl buffer (pH 1.2), acetate buffer (pH 4.5), and phosphate buffer (pH 6.8). Samples (5 mL aliquots) were withdrawn at 5, 10, 15, 20, 30, and 45 min, filtered through a 0.45‐μm membrane filter, and immediately replaced with an equal volume (5 mL) of fresh medium to maintain sink conditions; withdrawn samples were analyzed by the validated HPLC method (Section 2.2) [29].

Dissolution efficiency (DE), defined as the area under the dissolution curve up to time t, expressed as a percentage of the area of the rectangle representing 100% dissolution over the same time period, was calculated as

(1) DE%=∫0ty·dty100·t×100.

Mean dissolution time (MDT), the arithmetic mean time for drug release used to characterize release rate and compare formulations, was calculated as

(2) MDT=Σti·ΔMiΣΔMi,

where *t* *i* is the midpoint of each sampling interval and Δ*M*_*i*_ is the fraction of drug released during that interval. Dissolution profiles were compared using the model‐independent difference (*f*1) and similarity (*f*2) factors of Moore and Flanner, calculated using DDSolver; profiles were considered similar when *f*1 was between 0 and 15 and *f*2 was between 50 and 100 [30].

### 2.9. Ex Vivo Drug Absorption Study

The ex vivo permeability study was adapted with minor modifications from previously published study of SEDDS of gliclazide [31]. Fresh chicken small intestines were collected postmortem as discarded by‐products from a commercially licensed poultry facility immediately after slaughter (*Gallus gallus domesticus*). No animals were sacrificed or bred specifically for the purpose of this study. The duodenal portion of the small intestine was isolated and thoroughly washed with cold physiological saline (0.9% NaCl) to clear luminal contents and excess mucus and oxygenated. Intestinal segments of 3.0 cm in length (average internal diameter *d* = 0.6 cm) were prepared by securely tying one end of each segment with cotton thread (surface area 5.655 cm^2^).

A total of 500 mg of SMEDDS formulation, which is equivalent to 40 mg of ATV and 10 mg of EZE, was placed within the sac. A second sac containing 10 mg of pure EZE and 40 mg of ATV suspended in 450 mg of water was also used for comparison in this study.

Following that, the sacs were maintained at 37 ± 0.5 C, agitated at 100 rpm to ensure adequate hydrodynamic mixing and minimize static boundary layer resistance around the tissue membrane, and placed in different dissolution baskets with 900 cc of pH 6.8 phosphate buffer.

A calibrated plastic disposable syringe was used for collecting samples at predetermined intervals, and pure phosphate buffer was added each time. The withdrawn samples were filtered with a filter of a pore size of 0.45 μm. The HPLC method was used for analyzing the samples. All permeation experiments were performed in triplicate (*n* = 3) for each formulation group. Data are presented as mean ± standard deviation (SD).

### 2.10. Statistical Analysis

Unless otherwise stated, all experiments were performed as three independent replicates (*n* = 3); specific replicate numbers for each test are stated in the corresponding Methods subsection (Sections 2.3, 2.6.1–2.6.3, 2.9, 2.10). Data are presented as mean ± SD throughout. Statistical comparisons between formulations, and between formulation and reference/plain‐drug groups, were performed using one‐way analysis of variance (ANOVA) with Bonferroni‐corrected pairwise comparisons for comparisons involving more than two groups (e.g., solubility across oils, Section 3.2), or two‐sample *t* tests for single pairwise comparisons (e.g., ex vivo permeability, SMEDDS vs. plain drug, Section 3.11). Dissolution profile comparisons used the model‐independent difference (*f*1) and similarity (*f*2) factors of Moore and Flanner, calculated using DDSolver; profiles were considered similar when *f*1 was between 0 and 15 and *f*2 was between 50 and 100. Statistical analyses were performed using Microsoft Excel (built‐in statistical functions) and Python (SciPy library, version 1.x) for ANOVA, *t* tests, and post hoc pairwise comparisons. A significance threshold of *p* < 0.05 was applied throughout, with exact *p* values reported in the Results section.

## 3. Results

### 3.1. Development and Validation of the Analytical Method

The initial method adapted from the literature failed to resolve the EZE peak and produced an asymmetric ATV peak. Following method modification (Section 2.2), complete separation of both peaks was achieved, meeting all system suitability requirements (Figure 1).

**FIGURE 1:**
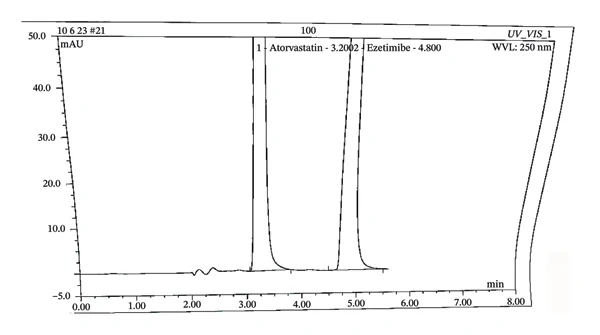
Chromatogram of the in‐house method developed to analyze the ATV/EZE combination.

Specificity testing showed no interference from the blank or placebo at the retention times of ATV or EZE (Table 3). System precision gave RSD values of 0.37% (ATV) and 0.27% (EZE) across six replicate injections, within the ≤ 2.0% acceptance limit. Accuracy (recovery) at 50%, 100%, and 150% of nominal concentration ranged from 98.1% to 100.7% (ATV) and 98.4%–100.9% (EZE), with %RSD ≤ 4.8% at all levels.

**TABLE 3:** Validation summary of the ATV/EZE analytical method.

Parameter	Acceptance criteria	Result	Conclusion
Specificity	Interference test: There should be no interference due to blank, placebo.	No interference observed	Confirm
	Forced degradation: degradation % from 5% to 20%	Ezetimibe: within target under peroxide (10.30%) and acid (10.20%); slightly below target under base (1.6%). Atorvastatin: exceeds target under peroxide (80.80%) and acid (80.70%); within target under base (10%). See Section 3.1 for full discussion; ATV peroxide/acid conditions to be repeated at reduced severity.	Confirm for EZE; partial for ATV (see 3.1)

Accuracy	Accuracy or % recovery at each concentration should be between 80 and 120.0 The % RSD for recovery of triplicate preparation for each level should not be more than 5.0%	% RSD of ATV: Level 1 (50%) = 3.8 Level 2 (100%) = 4.2 Level 3 (150%) = 1.5 % RSD of EZE: Level 1 (50%) = 4.7 Level 2 (100%) = 4.8 Level 3 (150%) = 3.7	Confirm
	System precision: the % relative standard deviation of the five standard NMT 2.0%	RSD is less than 1.0%	Confirm

Precision	Correlation coefficient is NLT 0.990.	*r* ^2^ Not independently assessed under precision; see Linearity and Range (*R* ^2^ = 0.9986 ATV/0.9984 EZE).	N/A—see Linearity and Range
	The relative standard deviation of the 10% variation in the flow rate, and UV wavelength should not be more than 2.0%	RSD Less than 2%	Confirm

Linearity and range	Accuracy or % recovery at each concentration should be between 80 and 120.0 The % RSD for the recovery of triplicate preparation for each level should not be more than 5.0%	% RSD of ATV: Level 1 (50%) = 3.8 Level 2 (100%) = 4.2 Level 3 (150%) = 1.5 % RSD of EZE: Level 1 (50%) = 4.7 Level 2 (100%) = 4.8 Level 3 (150%) = 3.7	Confirm
	System precision: The % Relative standard deviation of the five standard NMT 2.0%	RSD is less than 1.0%	Confirm
	Correlation coefficient is NLT 0.990.	*r* ^2^ of atorvastatin = 0.9986 *r* ^2^ of ezetimibe = 0.9984	Confirm
	The relative standard deviation of the 10% variation in the flow rate, and UV wavelength should not be more than 2.0%	RSD Less than 2%	Confirm

Robustness	Accuracy or % recovery at each concentration should be between 80 and 120.0 The % RSD for recovery of triplicate preparation for each level should not be more than 5.0%	% RSD of ATV: Level 1 (50%) = 3.8 Level 2 (100%) = 4.2 Level 3 (150%) = 1.5 % RSD of EZE: Level 1 (50%) = 4.7 Level 2 (100%) = 4.8 Level 3 (150%) = 3.7	Confirm
	System precision: The % relative standard deviation of the five standard NMT 2.0%	RSD is less than 1.0%	Confirm

Table 3 has been restructured to present each validation parameter (specificity, linearity, system/method precision, accuracy, robustness) once, in a single consolidated table, rather than the repeated/duplicated presentation of accuracy, precision, robustness, and linearity data noted in the original submission.

The calibration curves for both analytes were linear over the tested range, with correlation coefficients (*R* ^2^) of 0.9986 for ATV and 0.9984 for EZE, consistent with acceptable linearity per ICH Q2 (R2) (Figure 2). System suitability testing confirmed adequate chromatographic performance for both analytes (*n* = 6 replicate injections). Retention times were 3.19 ± 0.004 min for ATV and 4.81 ± 0.004 min for EZE (%RSD 0.12% and 0.08%, respectively). Peak resolution was 4.05–4.15, theoretical plate counts were 3560 (ATV) and 4250 (EZE), and tailing factors were 1.55 (ATV) and 1.26 (EZE)—all meeting the acceptance criteria stated in Section 2.2 (resolution ≥ 3.0, theoretical plates ≥ 2000, tailing factor 0.8–2.0). Solution stability was confirmed over 72 h at room temperature: peak areas for both sample and standard solutions of ATV and EZE remained within 0.12%–0.24% RSD across the 0, 24, 48, and 72 h timepoints, indicating no meaningful analyte degradation over this interval. The LOD and LOQ were determined from a dedicated calibration curve (50–200 ppm), calculated as 3.3 *σ*/S and 10 *σ*/S, respectively, from the residual standard deviation (σ) and slope (S) of that curve: LOD = 58.73 *μ*g/mL and LOQ = 177.98 *μ*g/mL for ATV; LOD = 53.98 *μ*g/mL; and LOQ = 163.57 *μ*g/mL for EZE.

**FIGURE 2:**
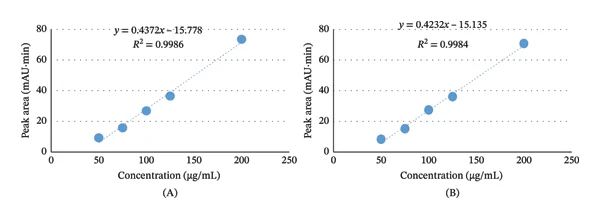
Standard calibration curve: (A) for atorvastatin and (B) for ezetimibe.

Forced degradation acceptance criteria required 5%–20% degradation under each stress condition, to confirm the method’s ability to resolve degradation products from the parent compounds without overdegrading the analyte beyond the point of reliable assessment. EZE fell within this target range under both acid (10.20% degradation) and peroxide (10.30% degradation) stress and was slightly below it under base stress (1.6% degradation). ATV, however, showed excessive degradation under both peroxide (80.80%) and acid (80.70%) stress and was within the target range under base stress (10%) (Table 2). The peroxide result reflects a repeat forced‐degradation run performed at an increased hydrogen peroxide concentration, undertaken to ensure degradation exceeded the originally specified (and, as discussed below, overly permissive) 20% minimum; this adjustment achieved that former criterion but over‐degraded ATV well beyond the revised 5%–20% target. Because so little parent ATV remained under peroxide and acid conditions, peak‐purity assessment relative to potential coeluting degradants was correspondingly limited for those two stress conditions; ATV’s specificity was adequately demonstrated only under base, heat, and UV stress, and EZE’s specificity was adequately demonstrated under all six conditions. We recommend that the ATV peroxide and acid stress conditions be repeated at reduced severity (e.g., a lower hydrogen peroxide concentration or shorter acid exposure time) in a future revalidation, to bring degradation into the 5%–20% target range and permit a reliable peak‐purity assessment.

### 3.2. Evaluation of ATV and EZE Solubility in Oils

Saturation solubility varied significantly across the seven oils tested (one‐way ANOVA: ATV *F* = 5140, *p* < 0.0001; EZE *F* = 327,491, *p* < 0.0001; Table 4). Triacetin gave the highest solubility for both drugs (ATV 8.07 ± 2.37 mg/mL, 95% CI 2.18–13.96; EZE 7.39 ± 0.30 mg/mL, 95% CI 6.65–8.14), significantly exceeding every other oil tested, including oleic acid, the second‐best performer (ATV 0.673 ± 0.03 mg/mL, 95% CI 0.60–0.75; EZE 0.062 ± 0.006 mg/mL, 95% CI 0.047–0.077). Triacetin’s solubility advantage over oleic acid was 12‐fold for ATV (*p* < 0.001) and 119‐fold for EZE (*p* < 0.0001; Bonferroni‐corrected pairwise *t* tests). We note the wide 95% CI for triacetin’s ATV solubility reflects the small sample size (*n* = 3) combined with relatively higher variability for that specific measurement; the statistical significance of the triacetin‐vs‐oleic acid difference is nonetheless robust; as the confidence intervals for the two oils do not overlap for either drug. Triacetin and oleic acid were carried forward for pseudoternary phase diagram construction.

**TABLE 4:** Saturation solubility of ATV/EZE in various oils (mean ± SD, *n* = 3).

Oil	ATV solubility (mg/mL)	EZE solubility (mg/mL)
Triacetin	8.07 ± 2.37	7.39 ± 0.30
Oleic acid	0.673 ± 0.03	0.062 ± 0.006
Soybean oil	0.146 ± 0.02	0.011 ± 0.002
Castor oil	0.055 ± 0.01	0.005 ± 0.001
Ethyl oleate	0.033 ± 0.01	0.034 ± 0.001
Olive oil	0.034 ± 0.01	0.008 ± 0.001
Sesame oil	0.027 ± 0.01	0.015 ± 0.001

*Note:* One‐way ANOVA *p* < 0.0001 for both drugs across all oils.

### 3.3. Pseudoternary Phase Diagrams and Formulation Screening

As can be seen in Figure 3a,b, pseudoternary phase diagrams were constructed for all systems that showed clear configuration—with the absence of the active materials—for all systems, employing: Forty‐five oil–surfactant–cosurfactant combinations were screened by water titration (Section 2.4); of these, formulations showing a clear, monophasic dispersion were carried forward to physical characterization. Acceptance criteria were droplet size 10–100 nm, PDI 0.1–0.7, refractive index 1.30–1.45, and viscosity 10–400 cP.

**FIGURE 3:**
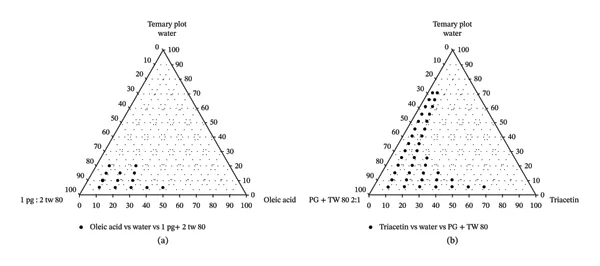
(a) Pseudoternary phase diagram of oleic acid + water + PG/TW 80 (1:2), (b) pseudoternary phase diagram of triacetin + water + PG/TW 80 (2:1).

To quantitatively evaluate the relative size of the microemulsion‐forming region for each oil, the percentage of water (w/w) that could be added before the onset of turbidity/phase separation was recorded for every screened trial and compared by oil type. Triacetin‐based systems tolerated significantly more water before phase separation (mean 43.1 ± 21.4% water, *n* = 26 trials across all surfactant/cosurfactant combinations tested) than oleic acid–based systems (mean 17.4 ± 4.7% water, *n* = 17 trials; two‐sample *t* test, *t* = 4.86, *p* < 0.0001; Table 1). This provides quantitative support for the larger microemulsion‐forming region observed for triacetin relative to oleic acid, rather than a visual/qualitative comparison of the phase diagrams alone.

Four formulations (ME#22, ME#26, ME#31, and ME#35) met the droplet size, PDI, refractive index, and viscosity criteria and were selected for further evaluation (Table 5). A fifth trial, ME#12 (oleic acid–based), is also shown in Table 1 for transparency but failed the PDI gating criterion: Its measured PDI (1.76 ± 0.92) not only falls outside the 0.1–0.7 acceptance range but exceeds the theoretical 0–1 range within which the cumulants‐based PDI calculation is physically defined, and a system with PDI approaching or exceeding 1.0 is more consistent with a coarse or phase‐separated emulsion than a microemulsion. We treat this result as indicating either a data‐recording anomaly or a genuinely nonmicroemulsion system for this trial, and, consistent with the gating rule stated above (formulations failing size/PDI criteria were excluded), ME#12 was excluded from formulation selection and from all subsequent characterization, stability, and dissolution testing; it is retained in Table 1 only as a documented rejected trial, not as a selected or retained comparator formulation. This corrects an earlier draft of this manuscript, which incorrectly described ME#12 as having been “retained” as a comparator despite failing PDI—a description that was inconsistent with the study’s own stated gating rule and has been removed.

**TABLE 5:** Formulas that met the physically accepted criteria.

ME#	Composition	Ratio oil: surf./co‐surf.	Ratio surf./co‐surf.	Viscosity (cp)	RI	Droplet size (nm)	Polydispersity
#T12	Oleic acid: Koll RH 40/PEG 400	1:9	1:1	300.2 ± 0.35	1.433	73.12 ± 8.64	1.76 ± 0.92
#T22	Triacetin: Tw 80/PG	2:8	2:1	12.31 ± 1.03	1.432	51.73 ± 2.97	0.35 ± 0.03
#T26	Triacetin: Tw 80/PG	3:7	3:1	244.35 ± 0.08	1.441	16.21 ± 2.03	0.07 ± 0.05
#T31	Triacetin: Tw 80/PEG 400	2:8	2:1	310 ± 9.58	1.372	74.15 ± 1.68	0.34 ± 0.02
#T35	Triacetin: Tw 20/PEG 400	2:8	1:1	65.85 ± 0.32	1.425	10.55 ± 2.72	0.17 ± 0.04

Selection criteria were applied hierarchically, not with equal weighting: droplet size and PDI were treated as primary/gating criteria, since these directly define whether a system qualifies as a microemulsion at all; refractive index served as a secondary confirmatory check for transparency/isotropy; viscosity was treated as a practical processability criterion (ensuring the formulation could be reliably pipetted and capsule‐filled, Section 2.7) rather than a primary selection driver and was not prioritized over droplet size in the selection logic—formulations failing the droplet size/PDI criteria were excluded regardless of viscosity. All physical characterization in this section (Table 6) was performed on the blank (drug‐free) formulations; active pharmaceutical ingredients were incorporated only after this screening stage, for the five selected formulations, for the subsequent solubility‐in‐microemulsion (Section 3.5), stability‐with‐actives (Section 3.7), and dissolution (Section 3.10) studies.

**TABLE 6:** Formulation selection matrix (blank formulations, 45 trials were screened).

ME#	Droplet size 10–100 nm	PDI 0.1–0.7	RI 1.30–1.45	Viscosity 10–400 cP	Selected?	Rationale
12	Pass (73.12)	FAIL (1.76)	Pass	Pass	No	Excluded—failed PDI gating criterion (PDI 1.76 ± 0.92; exceeds even the theoretical 0–1 range). Shown here for transparency as a documented rejected trial; not selected, retained, or used as a comparator formulation. Excluded from all subsequent characterization, stability, and dissolution testing.
22	Pass (51.73)	Pass (0.35)	Pass	Pass	Yes	Met all criteria
26	Pass (16.21)	Pass (0.07)	Pass	Pass	Yes	Met all criteria
31	Pass (74.15)	Pass (0.34)	Pass	Pass	Yes	Met all criteria; ultimately optimal on solubility/dissolution
35	Pass (10.55)	Pass (0.17)	Pass	Pass	Yes	Met all criteria; later excluded from stability/dissolution stage (Section 3.6) due to Tween 20 use and largest droplet growth on storage
Other 40 trials	Variable	Variable	Variable	Variable	No	Excluded for failing one or more gating criteria (typically droplet size/PDI), or biphasic/turbid at the tested oil:Smix ratio

### 3.4. Dye Miscibility Test

All five formulations showed uniform, homogeneous color distribution when tested with the water‐soluble dye methyl orange, without precipitation, confirming an oil‐in‐water (o/w) microemulsion configuration in each case.

### 3.5. Solubility of ATV/EZE in the Selected Microemulsion Formulations

Solubility differed significantly among the four selected formulations (one‐way ANOVA, recomputed excluding the excluded ME#12 trial: ATV *F* = 23.07, *p* = 0.0003; EZE *F* = 66.86, *p* < 0.0001; these values are corrected from an earlier draft, which had included ME#12 in this comparison despite its exclusion on PDI grounds). ME#31 gave the numerically highest combined drug solubility (ATV 3.95 ± 0.35 mg/mL; EZE 3.04 ± 0.06 mg/mL), followed by ME#22, ME#35, and ME#26 (Table 7). Pairwise comparison between ME#31 and its closest competitor, ME#22, showed a statistically significant difference for EZE (*t* = 4.23, *p* = 0.013) but not for ATV (*t* = 2.38, *p* = 0.076); we report this honestly rather than describing ME#31 as unambiguously superior on both drugs. For reference, the excluded ME#12 trial showed markedly lower solubility (ATV 0.53 ± 0.24; EZE 0.24 ± 0.14 mg/mL) than all four selected formulations, consistent with its exclusion on PDI grounds and providing additional context for why the oleic acid–based system was not carried forward.

**TABLE 7:** Solubility of ATV/EZE in selected microemulsion formulations (mean ± SD, *n* = 3).

ME#	ATV solubility (mg/mL)	EZE solubility (mg/mL)
31	3.95 ± 0.35	3.04 ± 0.06
22	3.28 ± 0.34	2.60 ± 0.17
35	2.73 ± 0.23	2.27 ± 0.02
26	2.11 ± 0.17	2.05 ± 0.02
12	0.53 ± 0.24	0.24 ± 0.14

### 3.6. Stability Studies for Formulations of Microemulsion (Without Active Ingredients)

ME#22, ME#26, ME#31, and ME#35 remained monophasic after the centrifugation stress test (Section 2.6.4), and droplet size (measured by DLS, Section 2.6.3) remained within the 10–100 nm acceptance range at time zero, after freeze–thaw cycling, and after one week at room temperature (Table 8). The 1‐week room‐temperature checkpoint was performed, in addition to the three‐stage thermodynamic stability protocol described in Section 2.6.4, as a short‐term stability check on formulations that had already passed heating‐cooling, centrifugation, and freeze–thaw stress testing, prior to committing them to drug loading; this additional step has been added explicitly to Section 2.6.4 for consistency between Methods and Results. Based on these results, ME#22, ME#26, and ME#31 were selected for stability testing with active ingredients and in vitro dissolution studies. ME#35, though physically stable, was excluded at this stage because its formulation used Tween 20 (rather than Tween 80, used in the other three), and it showed the largest relative droplet size change after one week of storage.

**TABLE 8:** Droplet size (nm) of blank microemulsion formulations at time zero, after freeze–thaw cycling, and after 1 week at room temperature (mean ± SD).

ME#	Time zero	After freeze–thaw	After 1 week (RT)
22	51.73 ± 5.29	21.35 ± 1.34	37.93 ± 2.97
26	16.21 ± 2.03	14.35 ± 0.26	11.51 ± 0.23
31	74.15 ± 1.68	62.58 ± 0.22	69.28 ± 1.27
35	10.55 ± 2.72	4.72 ± 0.40	4.26 ± 0.40

### 3.7. Stability Studies of the Selected Formulations of Microemulsion With Active Materials

ME#22, ME#26, and ME#31 (loaded with ATV/EZE) remained within acceptable assay limits (95%–107%) and particle size range (10–100 nm) at time zero, one week, and two weeks at room temperature (Table 9). One‐way ANOVA across the three timepoints showed no statistically significant change in assay for ATV in any formulation (ME#22: *F* = 4.99, *p* = 0.053; ME#26: *F* = 1.25, *p* = 0.352; ME#31: *F* = 3.91, *p* = 0.082), nor for EZE in ME#26 or ME#31 (*F* = 0.51, *p* = 0.626 and *F* = 1.75, *p* = 0.252, respectively). ME#22’s EZE assay showed a statistically significant change over the two‐week period (*F* = 7.03, *p* = 0.027); however, all EZE assay values for ME#22 remained within the 102%–107% acceptance range throughout, indicating this reflects normal assay variability rather than a meaningful degradation trend. This two‐week room‐temperature evaluation was designed as a rapid in‐process screening step to select among ME#22, ME#26, and ME#31 before committing a single optimized formulation (ultimately ME#31) to the more extensive, multicondition capsule‐level stability program (refrigerated/30°C/40°C for 30 days, Section 3.8) and the accelerated stress testing described in Section 3.7.1; it was not intended as a standalone or definitive stability claim, and we acknowledge two weeks falls short of standard pharmaceutical stability study durations.

**TABLE 9:** Assay (%) and particle size (nm) of drug‐loaded formulations at room temperature (mean ± SD).

ME#	Time point	ATV assay (%)	EZE assay (%)	Particle size (nm)	PDI
22	Time 0	103.5 ± 1.17	106.5 ± 1.42	68.34 ± 1.24	0.39 ± 0.75
22	Week 1	98.4 ± 3.36	105.31 ± 1.23	72.84 ± 0.37	0.27 ± 0.04
22	Week 2	99.57 ± 0.46	102.25 ± 1.62	68.05 ± 0.26	0.34 ± 0.12
26	Time 0	99.4 ± 0.58	102.9 ± 2.07	48.22 ± 0.59	0.07 ± 0.05
26	Week 1	99.06 ± 1.05	102.64 ± 1.75	34.52 ± 0.20	0.42 ± 0.24
26	Week 2	100.28 ± 1.19	103.87 ± 0.33	31.71 ± 0.41	0.28 ± 0.17
31	Time 0	104.6 ± 1.04	104.9 ± 0.91	53.21 ± 0.83	0.37 ± 0.02
31	Week 1	103.12 ± 1.65	103.22 ± 0.86	79.05 ± 0.31	0.25 ± 0.53
31	Week 2	100.71 ± 2.25	104.65 ± 1.63	67.38 ± 0.48	0.45 ± 0.53

#### 3.7.1. Accelerated Stability of Drug‐Loaded Formulations

In addition to the room‐temperature evaluation above, ME#22, ME#26, and ME#31 were also evaluated under accelerated stress conditions using the freeze–thaw and centrifugation protocols described in Section 2.6.4. All three formulations remained clear and monophasic after both freeze–thaw cycling and centrifugation, with no visible active precipitation, and assay values remained within acceptable limits after freeze–thaw cycling (Tables 10 and 11). A dedicated ICH‐guideline accelerated stability study (e.g., 40°C/75% RH for 6 months per ICH Q1A(R2)) was not performed in this work; the freeze–thaw and centrifugation stress tests reported here, together with the 30‐day elevated‐temperature capsule study (Section 3.8), provide preliminary accelerated‐condition data, but we acknowledge these do not substitute for a full ICH‐compliant accelerated stability study, which is recommended before regulatory submission.

**TABLE 10:** Assay (%) of drug‐loaded formulations after freeze–thaw cycling (mean ± SD).

ME#	Visual appearance	Actives precipitation	ATV assay (%)	EZE assay (%)
22	Clear, monophasic	No	98.24 ± 0.23	101.25 ± 2.61
26	Clear, monophasic	No	97.19 ± 0.51	99.31 ± 3.65
31	Clear, monophasic	No	96.57 ± 0.89	101.34 ± 3.47

**TABLE 11:** Visual appearance of drug‐loaded formulations after centrifugation stress testing.

ME#	Visual appearance	Actives precipitation
22	Clear, monophasic	No
26	Clear, monophasic	No
31	Clear, monophasic	No

### 3.8. Stability Study of Filled Capsules

The capsules have been subjected to a stability study at 30°C/65% RH, 40°C/75% RH and refrigerated conditions for a period of 1 month, with relative humidity controlled and confirmed at each condition (Section 2.8), and the results are presented in Tables 12, 13, and Figure 4. The density of the SMEDDS concentrate, determined gravimetrically (Section 2.7), was approximately 1 g/mL, corresponding to a fill volume of approximately 500 *μ*L per capsule for the target 500 mg fill weight.

**TABLE 12:** Results of stability studies of microemulsion filled in capsules.

Formula	*n*	Average content weight (mg)	SD (mg)	%RSD	Min (mg)	Max (mg)	Count exceeding ±7.5% limit	Count exceeding ±15% limit	Applicable Ph. Eur. limit	Result
ME #22	100	499.23	7.21	1.44%	472.50	517.10	0	0	±7.5% (avg mass ≥ 300 mg)	PASS
ME #26	100	499.74	10.29	2.06%	478.50	529.10	0	0	±7.5% (avg mass ≥ 300 mg)	PASS
ME #31	100	499.54	9.09	1.82%	479.00	520.90	0	0	±7.5% (avg mass ≥ 300 mg)	PASS
ME #35	100	498.90	11.95	2.39%	451.80	522.40	1	0	±7.5% (avg mass ≥ 300 mg)	PASS

*Note:* PASS/FAIL applies the Ph. Eur. 2.9.5 acceptance rule for the ≥ 300 mg category (±7.5%) to the full *n* = 100 sample per formula: No more than 2 units may exceed ±7.5% deviation from that formula’s average content mass, and none may exceed ±15%. The official test is specified for a sample of 20 units.

**TABLE 13:** The content of drug substance and visual appearance of the selected microemulsion formulations after 1‐month stability study.

Formula number	Formula composition	Ratio oil: surf/cosurf.	Ratio surf: cosurf.	Visual appearance	ATV assay % at 30°C	ATV assay %at refrigerator	EZE assay % at 30°C	EZE assay % at refrigerator
ME #22	Triacetin: Tw 80/PG	2:8	2:1	Clear	95.62 ± 0.16	95.10 ± 0.73	96.32 ± 0.26	98.57 ± 1.05
ME #26	Triacetin: Tw80/PG	3	3:1	Clear	95.66 ± 0.32	95.93 ± 1.21	99.54 ± 0.42	98.60 ± 2.13
ME #31	Triacetin: Tw 80/PEG 400	2:8	2:1	Clear	100.88 ± 0.25	100.69 ± 0.60	97.51 ± 1.04	97.59 ± 0.24
ME #35	Triacetin: Tw 20/PEG 400	2:8	1:1	Clear	98.46 ± 1.04	97.38 ± 0.51	97.12 ± 1.45	98.38 ± 0.67

**FIGURE 4:**
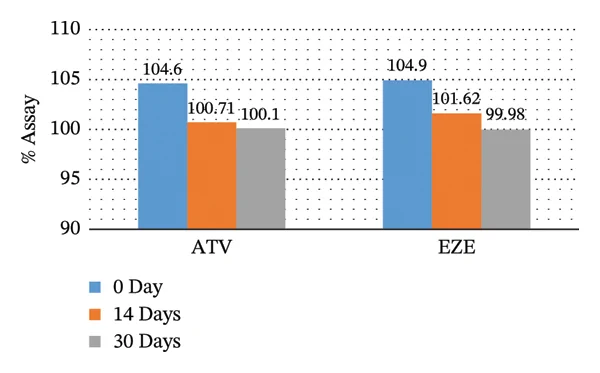
Content of drug substance of 31 formula capsules at conducted at 30°C/65% RH.

Fill weight uniformity across the filled capsules was assessed and found consistent with acceptable manual‐filling precision (with a mean fill weight of 499.54 mg, a SD of 9.09 mg, and a relative SD (%RSD) of 1.82% (*n* = 100 capsules tested per formula) (Table 12). Capsules stored at 30°C and under refrigeration retained ≥ 95% ATV and EZE content after 30 days, with no visible phase separation, indicating stability under these conditions. Capsules stored at 40°C developed leakage and could not be reliably assayed, indicating a formulation/packaging limitation at elevated temperature (Table 13).

The results of the stability study of samples stored at 30°C and at the refrigerator showed no remarkable change in the drug content, which remained above 95% for both of ATV and EZE, indicating that the combination is stable with no drug degradation at both of storage conditions; meanwhile, the capsules stored at 40°C suffered from leakage and could not be assayed.

### 3.9. In Vitro Drug Release Study

Formulations ME#22 (triacetin: Tween 80/PG 2:8), ME#26 (triacetin: Tween 80/PG 3:7), and ME#31 (triacetin: Tween 80/PEG 400 2:8) have been selected for assessing their dissolving performance based on the earlier findings. Formula ME#35 was excluded due to the fact that it had Tween 20, while the other formulations contained Tween 80, and it also had the largest droplet size after two weeks.

Figures 5A,B represent the dissolution profile comparison between the brand Atozet and formulas 22, 26, and 31, showing the dissolution profile at pH = 6.8.

**FIGURE 5:**
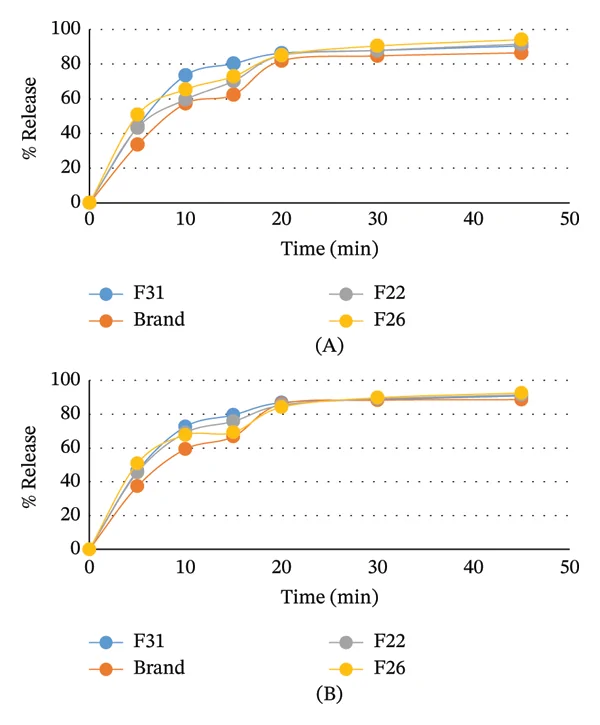
Dissolution profile of formulas 22, 26, and 31 vs. The brand at pH = 6.8, of (A) ezetimibe and (B) atorvastatin.

The dissolution parameters DE and MDT were calculated. As can be inferred from Table 14, the brand yielded the highest (slowest) MDT for both ATV and EZE, with values of 9.41 and 8.19 min, respectively (MDT is a time parameter and is corrected here from an earlier draft that incorrectly labeled these values with a percent sign). The brand also showed the lowest DE, with values of 68% and 72% for EZE and ATV, respectively. The similarity factor (F2) and difference factor (F1) were also calculated, and the results are listed in Table 14.

**TABLE 14:** Dissolution data of formulas 22, 26, 31 at pH = 6.8 for both ATV and EZE.

	Ezetimibe at pH = 6.8				Atorvastatin at pH = 6.8			
	Brand	F31	F22	F26	Brand	F31	F22	F26
MDT	9.41	8.27	9.08	8.65	8.19	6.81	7.93	8.09
DE	68%	74%	73%	76%	72%	77%	75%	75%
F2		56.00	61.23	50.00		53.00	64.53	59.00
F1		10	8	13		9	6	7

Formula 31 had the numerically lowest MDT and highest DE among the three formulations tested; however, as detailed below (Table 14, and the statistical comparison described in Section 3.10), one‐way ANOVA and post hoc pairwise testing did not show Formula 31 to be statistically distinguishable from Formula 22 or Formula 26 at most timepoints tested. This sentence has been corrected from an earlier draft, which stated Formula 31 had “the lowest MDT and highest percentage of drug release compared to other formulas” without statistical support and attributed this to the surfactant combination (Tween 80/PEG 400) as an established fact that mechanistic attribution is discussed later (Section 4.5) as a literature‐supported hypothesis rather than a demonstrated finding of this study, consistent with the statistical result that the three formulations were not significantly distinguishable from one another.

At pH 6.8, all three formulations released more drug than the Atozet brand product at every timepoint tested (Table 15). One‐way ANOVA comparing all four groups (ME#22, ME#26, ME#31, brand) at each sampling time showed statistically significant differences among groups at 5, 10, and 15 min for both drugs (*p* < 0.05 to *p* < 0.001), but not at 20, 30, or 45 min (*p* > 0.05 in all cases; Table 16), indicating the formulations and brand differ significantly in early release rate but converge to statistically indistinguishable extents of release by the later timepoints. Post hoc pairwise comparisons (Bonferroni‐corrected, *α* = 0.0083) at the significant early timepoints showed that each of ME#22, ME#26, and ME#31 individually differed significantly from the brand in earliest comparisons (e.g., ME#31 vs. brand: *p* = 0.0003 at 5 min and *p* = 0.0006–0.005 at 10–15 min for both drugs). However, ME#31 was not statistically distinguishable from ME#22 or ME#26 at any timepoint tested for either drug (all pairwise *p* > 0.05 after correction, with one exception: ME#26 vs. ME#31 for ATV at 15 min, *p* = 0.0066). This means that the statistically robust finding is that all three SMEDDS formulations released drug significantly faster than the brand product during the first 15 min of testing; the claim that ME#31 specifically is superior to ME#22 or ME#26 is not supported by statistically significant differences between these three formulations, and we have revised our characterization of ME#31 as “optimal” accordingly (Section 3.10, Discussion 4.5) to reflect its selection based on the combination of highest drug loading and favorable physicochemical properties (Sections 3.3, 3.5) rather than a statistically distinct dissolution advantage over ME#22/ME#26 specifically.

**TABLE 15:** Dissolution parameters at pH 6.8 (vs. brand).

Drug	Formula	MDT (min)	DE	*f*1	*f*2
EZE	Brand	9.41	0.68	—	—
EZE	F31	8.27	0.74	14	48
EZE	F22	9.08	0.73	8	61.23
EZE	F26	8.65	0.76	13	50
ATV	Brand	8.19	0.72	—	—
ATV	F31	6.81	0.77	9	53
ATV	F22	7.93	0.75	6	64.53
ATV	F26	8.09	0.75	7	58

**TABLE 16:** One‐way ANOVA (ME#22, ME#26, ME#31, brand) at each sampling time.

Drug	Time (min)	*F*	*p* value	Significant?
EZE	5	28.37	0.0001	Yes
EZE	10	9.18	0.0057	Yes
EZE	15	5.81	0.0208	Yes
EZE	20	1.48	0.291	No
EZE	30	2.84	0.105	No
EZE	45	3.44	0.072	No
ATV	5	11.13	0.0032	Yes
ATV	10	30.33	0.0001	Yes
ATV	15	15.57	0.0011	Yes
ATV	20	0.29	0.832	No
ATV	30	0.68	0.590	No
ATV	45	1.06	0.418	No

Formula 31 was selected for further evaluation across the physiologically relevant pH range (1.2, 4.5, 6.8), based on its combination of the highest drug solubility, favorable physicochemical profile, and fastest numerical in vitro release among the three formulations tested at pH 6.8 (Tables 17 and 18; see Section 3.10 regarding the statistical basis, and its limits, for formulation selection). At every pH tested, ME#31 showed a numerically lower MDT and higher DE than the brand product for both drugs. Individual replicate‐level data were not available for the pH 1.2 and 4.5 comparisons (only mean values were recorded for these media), so a timepoint‐by‐timepoint ANOVA/*t* test, of the kind reported for pH 6.8 in Table 16, could not be computed for these two media. The applicable formal statistical comparison for these full dissolution profiles is therefore the model‐independent difference (f1) and similarity (f2) factor analysis (Section 2.9, Section 2.11), which is specifically designed for comparing complete release profiles rather than individual timepoints. This analysis showed ME#31 was formally dissimilar from the brand at pH 1.2 and pH 4.5 for both drugs (*f*1 > 15 and/or *f*2 < 50 in all four comparisons (Tables 16 and 17); that is, despite the visual similarity in overall trend that may be apparent from the release curves (Figures 6 and 7), the f1/f2 analysis indicates the two profiles are not statistically equivalent at these pH values, consistent with ME#31 releasing more drug, more quickly, than the brand across the gastric and intestinal pH range tested.

**TABLE 17:** Dissolution parameters for ME#31 vs. brand across pH 1.2, 4.5, and 6.8.

Drug	Medium	*f*1	*f*2	Verdict
EZE	pH 6.8	14	48	Borderline dissimilar
ATV	pH 6.8	9	53	Similar
EZE	pH 1.2	51	40	Dissimilar
ATV	pH 1.2	52	58	Dissimilar (*f*1 high)
EZE	pH 4.5	20	47	Dissimilar
ATV	pH 4.5	20	49	Dissimilar

**TABLE 18:** Dissolution data for formula 31 at pH = 1.2, 4.5, and 6.8.

Time	Ezetimibe at pH = 6.8		Atorvastatin at pH = 6.8		Ezetimibe at pH = 4.5		Atorvastatin at pH = 4.5		Ezetimibe at pH = 1.2		Atorvastatin at pH = 1.2	
	F31	Brand	F31	Brand	F31	Brand	F31	Brand	F31	Brand	F31	Brand
MDT	7.27	9.40	6.81	8.19	10.28	10.39	14.48	14.52	3.85	5.13	9.70	10.16
DE	0.75	0.68	0.77	0.72	0.68	0.57	0.61	0.51	0.45	0.29	0.18	0.12
F2	48		53		47		49		40		58	
F1	14		9		20		20		51		52	

**FIGURE 6:**
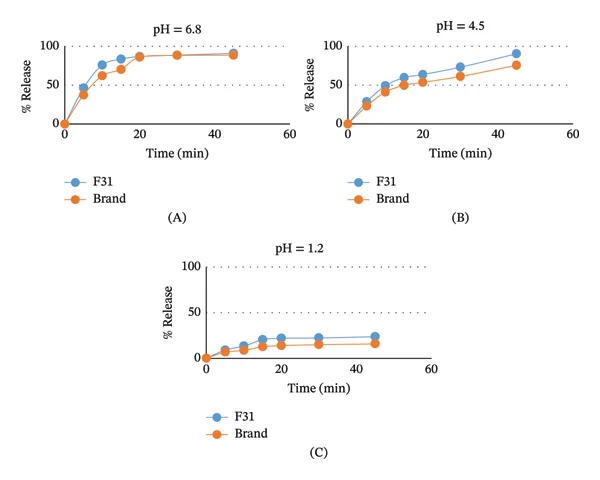
Dissolution profile of atorvastatin of formula 31 vs. brand: (A) at pH = 6.8, (B) at pH = 4.5, (C) at pH = 1.2.

**FIGURE 7:**
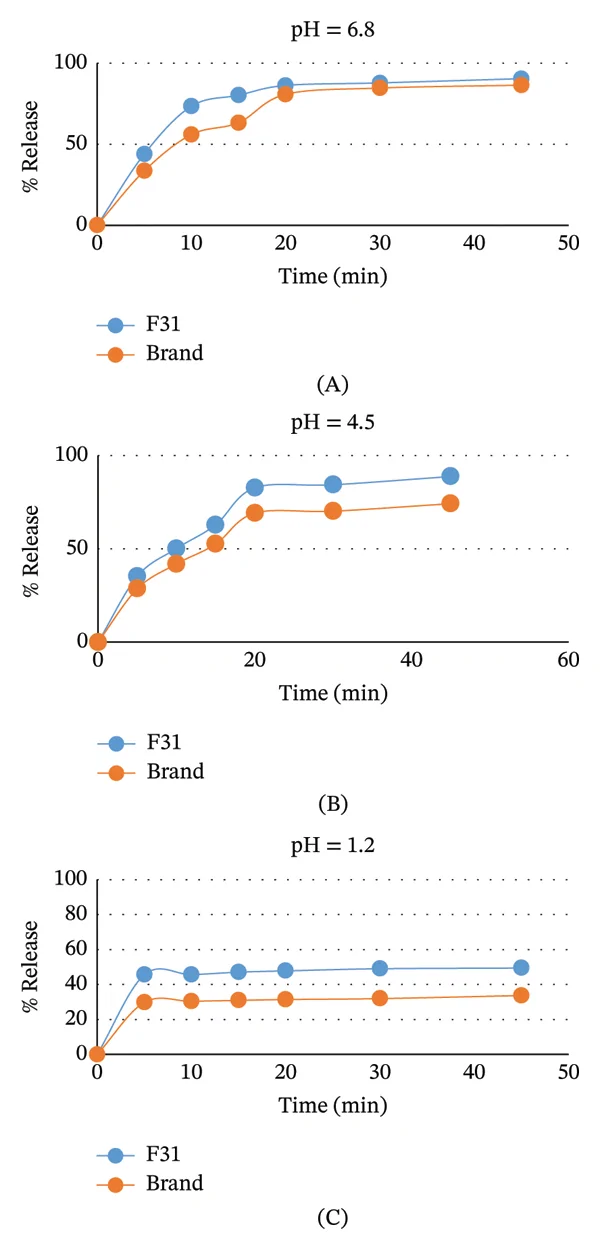
Dissolution profile of ezetimibe of the formula 31 vs. brand at (A) at pH = 6.8, (B) at pH = 4.5, (C) at pH = 1.2.

### 3.10. Ex Vivo Drug Permeability of ATV and EZE Through Chicken Intestinal Sac

In addition to the standard dissolving tests of SMEDDS, the ex vivo permeability of ATV and EZE from SEDDS via the chicken intestinal sac was investigated. There were no significant variations in the permeability profiles of ATV and EZE from the several used samples during the first hour, as shown in the graph. In contrast to samples containing the pure medicines, the SEDDS formulation showed improved absorption over time. However, SEDDS formulations showed above 95% diffusion within 5 h of the trial, whereas pure ATV and EZE only showed 75% and 67%, respectively (Figure 8).

**FIGURE 8:**
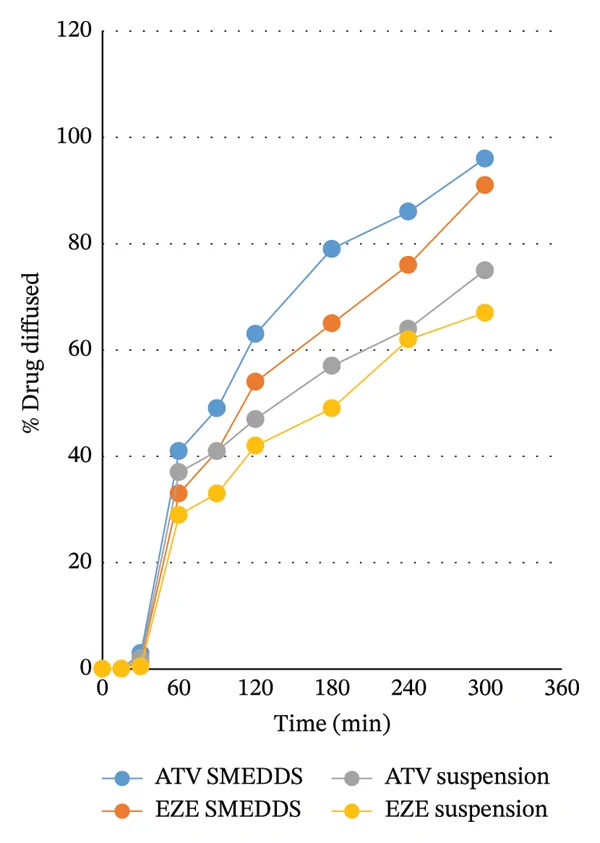
Permeability profile.

Observed onset of permeation is around 30 min (0% permeation at 15 min and with detectable permeation initiated at 30 min) prior to achieving steady‐state at 60 min. As for steady state fluxe (J) was obtained from the calculation of the slope of Qt/A (*μ*g/cm^2^) vs time (hours), where Qt is the cumulative amount of permeated drug and A the surface area. The permeability (Papp) calculated as Papp (cm/s) = J/(C0 X3600), where C_0_ is the donor initial concentration *μ*g/mL [32]. And the enhancement ratio was calculated as J (SMEDDS)/J (suspension) (Table 19).

**TABLE 19:** The results of permeability calculations.

Parameter	ATV SMEDDS	ATV suspension	EZE SMEDDS	EZE suspension
Observed lag time (*t*_lag_)	∼30 min	∼30 min	∼30 min	∼30 min
Steady‐state flux (*J*_*s* *s*_)	970.94 *μ*g/cm^2^/h	665.66 *μ*g/cm^2^/h	247.95 *μ*g/cm^2^/h	174.23 *μ*g/cm^2^/h
Linearity (*R* ^2^, 60 − −300 min)	0.9570	0.9963	0.9859	0.9819
*P*_app_ (10^−6^ cm/s)	3.37 × 10^−6^	2.31 × 10^−6^	3.44 × 10^−6^	2.42 × 10^−6^
Enhancement ratio (ER)	1.46	—	1.42	

The SMEDDS formulation (ME#31) showed significantly greater cumulative drug diffusion across the chicken intestinal sac than plain drug suspension for both actives, with the difference reaching statistical significance from 90 min onward (two‐sample *t* test, *n* = 3, *p* < 0.05; Table 20). At 5 h, the SMEDDS formulation showed 96 ± 3.5% (ATV) and 91 ± 5.1% (EZE) cumulative diffusion, compared with 75 ± 4.0% (ATV) and 67 ± 5.2% (EZE) for the plain drug suspension. This differs from an earlier draft of this manuscript, which included an ambiguously worded statement about “several used samples during the first hour”; that statement has been removed and replaced with the explicit sampling description above, since the two‐sample *t* test results in Table 19 already show no statistically significant difference between SMEDDS and plain suspension specifically at the 60‐min timepoint (*p* = 0.059 for ATV, *p* = 0.097 for EZE)—which is the likely basis for the original, unclear statement about the first hour.

**TABLE 20:** Ex vivo permeability of ATV/EZE: SMEDDS vs. plain drug suspension (mean % diffused ± SD, *n* = 3).

Time (min)	ATV SMEDDS	ATV plain	*p* value	EZE SMEDDS	EZE plain	*p* value
60	41 ± 1.6	37 ± 2.1	0.059	33 ± 2.0	29 ± 2.5	0.097
90	49 ± 1.75	41 ± 4.5	0.046^∗^	41 ± 1.55	33 ± 3.6	0.024^∗^
120	63 ± 4.3	47 ± 5.3	0.015^∗^	54 ± 3.5	42 ± 3.5	0.014^∗^
180	79 ± 3.4	57 ± 5.2	0.004^∗∗^	65 ± 4.6	49 ± 4.7	0.014^∗^
240	86 ± 4.4	64 ± 4.6	0.004^∗∗^	76 ± 5.2	62 ± 3.8	0.020^∗^
300	96 ± 3.5	75 ± 4.0	0.002^∗∗^	91 ± 5.1	67 ± 5.2	0.005^∗∗^

^∗^ *p* < 0.05.

^∗∗^ *p* < 0.01.

## 4. Discussion

### 4.1. Evaluation of ATV and EZE Solubility

The markedly higher solubility of both drugs in triacetin compared with the other oils tested (12‐fold and 119‐fold higher than oleic acid for ATV and EZE, respectively; Section 3.2) may be attributed to triacetin’s amphiphilic character: As a short‐chain triglyceride bearing three ester groups, it combines sufficient hydrophilic character to act as a cosolvent with the ability to solubilize lipophilic drug molecules [33, 34].

Oleic acid gave the second‐highest solubility for both drugs. This may be related to interaction between its carboxylic acid group and the active ingredients; however, as no complementary analytical technique (e.g., FTIR, DSC) was used to confirm complex formation in this study, this explanation should be regarded as a plausible mechanism rather than a confirmed finding [35, 37].

### 4.2. Construction of Pseudoternary Phase

Triacetin produced significantly larger microemulsion regions than oleic acid, quantitatively confirmed by the substantially greater water tolerance observed before phase separation across all screened trials (43.1% vs. 17.4% water, *p* < 0.0001; Section 3.3), consistent with its higher HLB and greater compatibility with the aqueous phase, whereas oleic acid’s low HLB value (1.0) likely limited its emulsification capacity. A broader microemulsion region was also observed at higher surfactant‐to‐cosurfactant (Smix) ratios, consistent with increased surfactant adsorption at the interface and reduced interfacial tension [37]. Formulations with oil content exceeding 25% (w/w) showed phase separation, likely due to oil droplet coalescence at higher oil loading; the smallest droplet sizes were observed at 5%–10% (w/w) oil, with approximately 15% (w/w) identified as the practical optimum (Figure 9).

**FIGURE 9:**
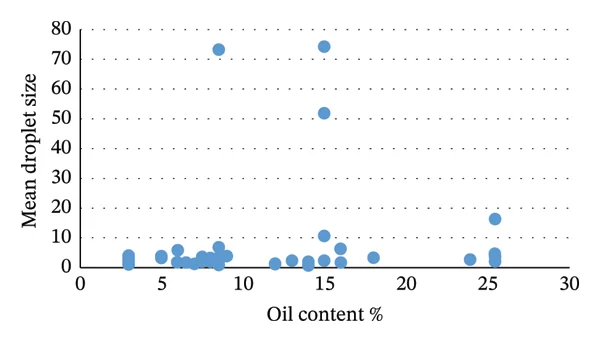
Effect of the oil content on the mean droplet size of SMEDDS.

Note on droplet size and oil type: Table 1 shows that droplet size did not track oil type in a simple way—the oleic acid–based formulation (ME#12: 73.12 nm) did not produce a smaller droplet than all triacetin‐based formulations; ME#26 (16.21 nm) and ME#35 (10.55 nm), both triacetin‐based, were substantially smaller than ME#12. Droplet size in this dataset therefore appears to depend more on the surfactant/cosurfactant system and Smix ratio used within each oil than on oil identity alone.

### 4.3. Characterization and Evaluation Tests

PDI is theoretically bounded between 0 and 1 under the cumulants‐based calculation used in this study (Section 2.6.3); values across the four selected formulations ranged from 0.07 to 0.45, within this theoretical range. Formulations with PDI between 0.1 and 0.7 were considered homogeneous with an acceptably narrow size distribution and were selected for further testing (ME#22, ME#26, ME#31, ME#35). The rejected ME#12 trial returned a PDI of 1.76, which exceeds the theoretical 0–1 range and is therefore not interpretable as a physically valid polydispersity value under the cumulants method; a value at or above 1.0 is more consistent with a coarse emulsion or phase‐separated system than a true microemulsion. Consistent with the study’s stated gating rule, ME#12 was excluded from selection and from all further testing on this basis (Section 3.3); it was not retained as a comparator formulation, correcting an inconsistent description in an earlier draft of this manuscript.

Droplet size cutoffs for microemulsions vary considerably across the literature, with different sources citing ranges as narrow as 5–50 nm and as broad as 10–400 nm; there is no single, universally accepted numerical boundary. A recent review specifically addressing this terminology confusion notes that microemulsion/nanoemulsion misclassification is increasingly common in the pharmaceutical literature and that the more mechanistically rigorous distinguishing criterion is thermodynamic stability and spontaneous formation (characteristic of true microemulsions) versus kinetic stability requiring high‐shear energy input for formation (characteristic of nanoemulsions), rather than droplet size alone [38]. By this criterion, the systems in this study are consistent with microemulsion classification: They form spontaneously upon gentle agitation/dilution, without high‐shear homogenization, sonication, or other externally applied mechanical energy—the defining mechanistic feature of a true (thermodynamically stable) microemulsion, and the basis of the “self‐microemulsifying” terminology used throughout [39].

A methodological limitation should be noted regarding the particle size measurements reported throughout this study (Section 2.6.3): DLS was performed on the neat SMEDDS concentrate rather than on the oil‐in‐water microemulsion formed upon aqueous dilution, and no dilution protocol was used at any stage of this measurement. Conventionally, SMEDDS droplet size is reported for the diluted system, since this reflects the dispersed state the formulation adopts upon contact with gastrointestinal fluid, and is the parameter most directly linked to interfacial surface area and absorption potential in the literature; we were unable to identify a precedent in the SMEDDS/microemulsion literature for reporting droplet size on an undiluted concentrate. This distinction is not purely conventional: DLS theory, including the Stokes–Einstein relationship used to derive particle size, formally applies to dilute systems, and instrument manufacturers caution that measurements on overly concentrated samples are subject to multiple scattering, in which light scattered by one particle is rescattered by neighboring particles before reaching the detector—an effect that characteristically causes the measured particle size to appear artificially small rather than simply noisier [40]. Given the SMEDDS concentrate’s relatively high particle/aggregate density prior to dilution, we cannot rule out some contribution from multiple scattering to the reported values, meaning that the true particle/aggregate size in the neat system may be somewhat larger than reported. The values reported here therefore more accurately reflect the size of surfactant‐associated nanostructures (e.g., reverse micelles or similar aggregates) within the concentrated, largely anhydrous system, rather than the microemulsion droplet size that would form in vivo. While these measurements were nonetheless useful for relative comparison and screening between formulations, and the resulting size ranges (10–100 nm) are broadly consistent with values reported for microemulsion droplets in the literature, we did not independently verify that the neat‐system particle size and the postdilution droplet size are equivalent for this system and recommend that future work include droplet size determination on the aqueous‐diluted formulation—diluted at a ratio consistent with the wide range reported for comparable SMEDDS systems (1:10 to 1:1000 SMEDDS:water, with 1:100 most commonly used, and 1:1000 reported specifically for an ATV calcium SMEDDS)40—together with reporting of scattering angle and equilibration time (not recorded for the present measurements), to directly confirm the in vivo–relevant dispersed particle size. Separately, we note that the ME#12 trial’s PDI value (1.76) exceeds not only the 0.1–0.7 acceptance range but the theoretical 0–1 range within which the cumulants‐based PDI calculation is physically defined; this value is not interpretable as a valid polydispersity measurement and instead indicates either a data‐recording anomaly or a system that has undergone phase separation or coarse emulsification rather than forming a true microemulsion, consistent with our treatment of this trial as excluded from formulation selection (Section 3.3) rather than as a retained comparator.

A limitation in continuous‐phase identification is that dye miscibility testing was the sole method used to confirm the oil‐in‐water (o/w) configuration of formulations. Although this test provided clear results, it is not definitive on its own; electrical conductivity measurement, which shows higher conductivity in o/w systems, is the standard complementary method and was not conducted in this study. However, the formulation approach—utilizing only hydrophilic, high‐HLB surfactants and focusing on monophasic, clear, low‐viscosity dispersions—helps mitigate uncertainty, as only o/w‐forming regions were selected [41]. Conductivity measurement is still recommended for future research as a confirmatory method.

### 4.4. Solubility Studies

ME#31 (triacetin 14.96%, Tween 80 39.89%, PEG 400 19.94%) showed the numerically highest combined drug loading among the five formulations, statistically significant for EZE but not for ATV specifically when compared against its closest competitor, ME#22 (Section 3.5). Comparing ME#31 and ME#35—identical oil and cosurfactant, differing only in surfactant (Tween 80 vs. Tween 20) and surf:cosurf ratio (2:1 vs. 1:1)—the higher Tween 80 proportion in ME#31 corresponded with both higher drug loading and a more favorable droplet size, suggesting a 2:1 surf:cosurf ratio with Tween 80 provided more effective self‐microemulsification than the 1:1 ratio with Tween 20 in this system.

Comparing ME#22 and ME#26—identical excipients but differing oil:Smix (2:8 vs. 3:7) and Smix (2:1 vs. 3:1) ratios—both formulations performed similarly, suggesting that the system was relatively tolerant to this range of ratio adjustment [2].

Regarding the choice of cosurfactant, this dataset allows a data‐grounded (if not fully independently controlled) comparison between PG (used in ME#22, ME#26) and PEG 400 (used in ME#31, ME#35), both paired with triacetin and a Tween‐series surfactant. The PEG 400–containing formulations showed a somewhat higher average combined drug solubility (ATV 3.34, EZE 2.66 mg/mL, mean of ME#31/ME#35) than the PG‐containing formulations (ATV 2.70, EZE 2.33 mg/mL, mean of ME#22/ME#26; Section 3.5, Table 5). A plausible explanation is PEG 400’s higher molecular weight and greater hydrophilic chain length relative to PG, which may improve its ability to occupy the interfacial region alongside Tween‐series surfactants and modestly increase the interfacial loading capacity for both drugs. We note, however, that cosurfactant identity was not varied independently of surfactant type and Smix ratio in this screening (Table 5), so this comparison should be interpreted as an associative observation from the available formulations rather than a controlled, mechanistically isolated finding; a factorial screening varying cosurfactant identity alone would be needed to confirm this effect.

Tween 80 has been reported to inhibit P‐glycoprotein (P‐gp) efflux and hepatic/intestinal CYP3A4 activity in other systems, which may contribute to improved bioavailability of P‐gp/CYP3A4 substrates such as ATV; however, this mechanism was not directly tested in the present study and is presented here as a literature‐supported hypothesis rather than a finding of this work, consistent with recent reviews of pharmaceutical excipients as P‐gp inhibitors [16, 42].

### 4.5. In Vitro Drug Release Study

All three SMEDDS formulations (ME#22, ME#26, ME#31) released significantly more drug than the Atozet brand product during the first 15 min of dissolution testing (one‐way ANOVA *p* < 0.05 to *p* < 0.001 at 5, 10, and 15 min; Section 3.10), with differences from the brand no longer statistically significant by 20–45 min as all groups converged toward comparable extents of release. Among the three formulations themselves, however, pairwise comparisons did not distinguish ME#31 from ME#22 or ME#26 at any timepoint for either drug (with one exception at 15 min for ATV). ME#31 was selected as the lead formulation on the basis of its highest combined drug loading (Section 3.5) and favorable physicochemical profile (droplet size, PDI, viscosity; Table 5) rather than a demonstrated, statistically significant dissolution advantage over ME#22 or ME#26 specifically.

This faster early release relative to the brand, common to all three SMEDDS formulations, is likely multifactorial rather than attributable to Tween 80 alone, and several specific physicochemical mechanisms plausibly contribute. First, interfacial phenomena: At Tween 80 concentrations of 30%–40% w/w (as used in ME#22, ME#26, and ME#31; Table 1), surfactant packing at the oil–water interface would be expected to approach saturation, which may lower interfacial tension substantially and favor spontaneous curvature toward small, kinetically accessible oil‐in‐water structures upon aqueous dilution—consistent with the sub‐100‐nm particle sizes measured for these formulations (Section 2.6.3, with the measurement‐context caveat discussed in Section 4.3) and with faster interfacial mass transfer once the SMEDDS contacts the dissolution medium [43, 44]. Second, molecular interactions: Both ATV and EZE are highly lipophilic (logP ∼4.1 and ∼4.5, respectively) and would be expected to partition preferentially into the surfactant‐rich interfacial region and triacetin‐rich core of the self‐emulsified droplets rather than existing as separate crystalline particles, avoiding the crystal lattice energy that would otherwise need to be overcome for a solid dosage form to dissolve—the drugs may therefore be effectively presolubilized at the moment of contact with dissolution medium, in contrast to the brand tablet, where dissolution requires both disintegration and subsequent crystalline dissolution [44, 45]. Third, droplet stabilization and surface area: The small droplet size conferred by the Tween 80/triacetin/PEG 400 or PG combination (Table 5) would be expected to increase the total interfacial surface area available for drug release per unit dose, which could contribute to a higher rate of diffusion‐limited mass transfer as predicted by the Noyes–Whitney relationship [21]. Formulation composition differences among ME#22, ME#26, and ME#31 (Smix ratio, oil:Smix ratio; Table 5) did not translate into statistically distinguishable dissolution rates among themselves (above), suggesting that these three specific compositions, while differing somewhat in droplet size and viscosity, may fall within a broadly similar functional regime for interfacial surfactant packing and resulting release rate, even though they differ measurably in drug‐loading capacity (Section 3.5). Self‐emulsification efficiency and interfacial tension were not directly quantified in this study and would strengthen these mechanistic interpretations if measured in future work (e.g., via pendant drop tensiometry or emulsification time/droplet size kinetics upon dilution).

A related point concerns the ionization behavior of EZE, which is more appropriately discussed here than as a standalone Results statement. EZE is weakly acidic by virtue of a phenolic hydroxyl group (pKa 9.75)—not nonionizable, as stated in an earlier draft of this manuscript, nor basic. However, because this pKa lies well above the pH range relevant to oral absorption (gastric pH ∼1.2 through intestinal pH ∼6.8–7.4, all tested in this study; Section 3.10), EZE remains predominantly unionized (> 99%) throughout the physiologically relevant gastrointestinal pH range and only becomes appreciably ionized at pH values well above 9, which are not physiologically encountered during oral absorption [46]. This is consistent with EZE’s comparably poor aqueous solubility across all three dissolution media tested (pH 1.2, 4.5, and 6.8; Table 11)—unlike a drug with a pKa within the physiological range, EZE’s dissolution behavior is not expected to, and did not, show pH‐dependent solubility enhancement across this range, since its ionization state does not meaningfully change across it [47].

### 4.6. Ex Vivo Drug Permeability of ATV and EZE Through Chicken Intestinal Sac

The higher ex vivo permeability observed for the SMEDDS formulation relative to plain drug suspension, which was statistically significant from 90 min onward (*p* < 0.05, two‐sample *t* test; Section 3.11), may reflect several contributing factors. Both ATV (logP ∼4.1) and EZE (logP ∼4.5) have low aqueous solubility, which likely limits dissolution‐dependent absorption from plain suspension; by contrast, molecular dispersion of both drugs within microemulsion droplets may maintain a higher apparent concentration at the absorptive surface and reduce precipitation in intestinal fluid, consistent with mechanisms reported for other SMEDDS systems. The larger interfacial surface area associated with sub‐100‐nm droplets could also increase drug–membrane contact. Surfactants and cosurfactants used in SMEDDS formulations have additionally been reported to transiently affect membrane permeability and to inhibit efflux transporters (P‐gp) in other systems; however, as neither membrane permeabilization nor P‐gp/CYP inhibition was directly assessed in this study, these remain plausible contributing mechanisms drawn from the literature rather than conclusions supported by the present data [27, 42].

### 4.7. Synthesis and Overall Significance

Taken together, these findings indicate that formulating ATV and EZE as a triacetin‐based SMEDDS meaningfully improves early dissolution rate relative to the marketed Atozet tablet (Section 3.10, Section 4.5) and significantly enhances ex vivo intestinal permeability relative to plain drug suspension (Section 3.11, Section 4.6), while several aspects of the underlying physical characterization (droplet size measurement conditions, continuous‐phase confirmation, thermodynamic stability testing) would benefit from further confirmatory work before the formulation’s classification and performance claims are considered fully established (Section 4.3).

Our solubility findings are broadly consistent with, and in one respect closely quantitatively corroborated by, prior work on ATV/EZE SMEDDS systems. Hwang et al. reported an optimized SMEDDS oil‐phase solubility of 7.87 mg/mL for ATV, essentially identical to the 8.07 mg/mL we obtained for ATV in triacetin (Section 3.2) despite different oil/surfactant systems being used, which lends independent, cross‐study support to the magnitude of solubility enhancement achievable for ATV in a well‐optimized SMEDDS oil phase [20]. By contrast, the same study reported a considerably higher EZE solubility (203.29 mg/mL) than we observed for EZE in any oil tested in this work (maximum 7.39 mg/mL in triacetin), a roughly 27‐fold difference that likely reflects their use of a different, EZE‐optimized oil/surfactant combination rather than a limitation of the water‐titration screening method itself, and highlights that oil selection remains a highly system‐specific optimization rather than one with a universal best choice across studies. Similarly, Hwang et al. [20] reported qualitatively faster initial dissolution for their SMEDDS relative to marketed product, consistent with our own early timepoint dissolution findings (Section 3.10), reinforcing that this is a reproducible feature of ATV/EZE SMEDDS formulations across independent studies rather than an isolated result specific to this work.

Our reported particle sizes (10–74 nm across the five screened formulations, Table 1) are, on their face, smaller than droplet sizes reported for other published ATV‐containing SEDDS/SMEDDS systems—for example, 198.4 nm reported for a solidified ATV S‐SMEDDS[48], 165.3 nm for a dispersible ATV self‐microemulsifying tablet [49], and 145–967 nm across nine SMEDDS formulations of resveratrol, a comparably lipophilic compound [50]. However, given the droplet‐size measurement limitation already discussed (Section 4.3)—our values were measured on the neat, undiluted SMEDDS concentrate rather than the postdilution microemulsion, unlike the standard practice in these comparator studies—this apparent size advantage should not be over‐interpreted as a demonstrated superiority; it may equally reflect the different measurement approach rather than a genuinely smaller in vivo–relevant droplet size, reinforcing the priority of confirming droplet size on the diluted system in follow‐up work. One directionally consistent finding across studies is worth noting: A prior ATV SEDDS study similarly reported that increasing surfactant content decreased droplet size while increasing dissolution rate [51], the same qualitative relationship we infer from the higher‐Tween‐80 formulations in this work (Section 4.4–4.5), which lends some independent support to this compositional trend even though our own droplet size data carry the measurement caveat noted above.

The principal limitations of this study, considered together rather than in isolation, are as follows: reliance on droplet size measurements taken on the undiluted SMEDDS concentrate rather than the postdilution microemulsion (Section 4.3); a single confirmatory method (dye miscibility) for continuous‐phase identification (Section 4.3); statistical comparisons among the three candidate formulations (ME#22, ME#26, ME#31) that did not reach significance at most dissolution timepoints, meaning formulation selection rested more on physicochemical profile and drug loading than on a demonstrated dissolution advantage (Section 4.5); and the absence of in vivo pharmacokinetic data to confirm that the observed in vitro and ex vivo advantages translate into improved systemic bioavailability. None of these limitations undermine the core, statistically supported findings of this work—that triacetin‐based SMEDDS formulations solubilize ATV and EZE far more effectively than the oils conventionally used for this drug pair and that the resulting formulations release drug faster than the marketed product and permeate intestinal tissue more effectively than plain drug suspension—but they do mean the formulation should be regarded as a promising early stage candidate requiring further characterization and in vivo confirmation, rather than a finished, fully validated delivery system.

## 5. Conclusions

An optimized formulation of ATV‐/EZE‐loaded SMEDDS encapsulated in a soft gel capsule was successfully developed. The formulation contained 10 mg EZE, 40 mg ATV, 14.96% triacetin (oil), 39.89% Tween 80, and 19.94% PEG 400. This formula produced a stable microemulsion when exposed to an aqueous medium, despite changes in pH levels. The optimized formulation demonstrated enhanced in vitro dissolution and ex vivo permeability, suggesting the potential for improved oral bioavailability. However, these findings should be confirmed through appropriate in vivo pharmacokinetic and bioavailability studies.

## Author Contributions

Conceptualization, Yara Nabeel Al‐Salhi, Hani Naseef, Abdullah K. Rabba, and Moammal Qurt; methodology, Yara Nabeel Al‐Salhi, Hani Naseef, Abdallah Damin Abukhalil, and Moammal Qurt; analytical method validation, Yara Nabeel Al‐Salhi and Fuad Al‐Rimawi; formal analysis, Yara Nabeel Al‐Salhi and Israr Sabri; data curation, Yara Nabeel Al‐Salhi and Israr Sabri; writing–original draft preparation, Yara Nabeel Al‐Salhi and Hani Naseef; writing–review and editing, Hani Naseef, Abdullah K. Rabba, Abdallah Damin Abukhalil, Moammal Qurt, and Fuad Al‐Rimawi; supervision and Moammal Qurt; project administration, Hani Naseef and Moammal Qurt; Please turn to the CRediT taxonomy for the term explanation. Authorship must be limited to those who have contributed substantially to the work reported.

## Funding

This research was funded by the Faculty of Graduate Studies and Research at Birzeit University.

## Disclosure

All authors have read and agreed to the published version of the manuscript.

## Ethics Statement

The authors have nothing to report.

## Consent

The authors have nothing to report.

## Conflicts of Interest

The authors declare no conflicts of interest.

## Data Availability

The data that support the findings of this study are available from the corresponding author upon reasonable request.
